# Numerical analysis of the electrical failure of a metallic nanowire mesh due to Joule heating

**DOI:** 10.1186/1556-276X-8-370

**Published:** 2013-08-30

**Authors:** Yuan Li, Kaoru Tsuchiya, Hironori Tohmyoh, Masumi Saka

**Affiliations:** 1 Department of Nanomechanics, Tohoku University, Aoba 6-6-01, Aoba-ku, Sendai 980-8579, Japan

**Keywords:** Electrical failure, Joule heating, Metallic nanowire mesh, Stable/unstable melting

## Abstract

To precisely examine the electrical failure behavior of a metallic nanowire mesh induced by Joule heating (i.e., melting), a previously developed numerical method was modified with regard to the maximum temperature in the mesh and the electrical resistivity of the nanowire. A sample case of an Ag nanowire mesh under specific working conditions was analyzed with highly accurate numerical results. By monitoring the temperature in the mesh, the current required to trigger the melting of a mesh segment (i.e., the melting current) could be obtained. The melting process of a mesh equipped with a current source during actual operation was predicted on the basis of the obtained relationship between the melting current and the corresponding melting voltage in the numerical melting process. Local unstable and stable melting could be precisely identified for both the current-controlled and voltage-controlled current sources in the present example.

## Background

Given the remarkable physical properties of metallic nanowires, their successful assembly into both regular [1,2] and random [3-5] networks can achieve three goals: high transparency, low electrical resistivity, and good flexibility. Therefore, these metallic nanowire networks offer promising alternatives to indium tin oxide (ITO) for possible application in optoelectronic devices, such as touch screens and solar cells. For example, high optical transmittance and electrical conductance have been reported for a flexible transparent Cu nanowire mesh (i.e., a regular network) [1]. In addition, an organic solar cell integrated with such a Cu nanowire mesh electrode has been shown to perform comparably to one using an ITO electrode [1]. Another study on a transparent conductive Ag nanowire mesh has also been shown to exhibit a similarly good performance [2].

As we all have known, when current flows through any electrically conductive material, some electrical energy is transformed into thermal energy, which means the occurrence of Joule heating [6]. Undoubtedly, this general knowledge also applies to individual metallic nanowire and the corresponding nanowire mesh, both of which are conductors. Due to the size effects on the nanoscale (e.g., the increase in electrical resistivity [7-9] and the decrease in both thermal conductivity [10-12] and melting point [13,14]), the high current density and the substantial Joule heating induced in metallic nanowires may cause or accelerate electrical failure related to the phenomena of melting [15-17], electromigration [16,18-21], and corrosion [22]. The size effects will definitely also degrade the electrical performance of the corresponding nanowire mesh and therefore reduce the reliability of mesh-based devices. To prevent this problem, there is an urgent need to examine the electrical failure of a metallic nanowire mesh induced by Joule heating.

Unfortunately, in contrast with the numerous reports on electrical failure of individual metallic nanowires [15-21], little is currently known about the electrical failure of metallic nanowire mesh, which is expected to exhibit different characteristics because of its unique mesh structure. A recent and pioneering study [23] reported the electrical failure of an Ag nanowire random network due to Joule heating and offered possible solutions to the potential for electrical failure of a metallic nanowire mesh. In addition, a numerical method has also been proposed [24] which provided meaningful yet preliminary results regarding the electrical failure of a metallic nanowire mesh due to Joule heating.

The present work aims to clarify the electrical failure behavior of a metallic nanowire mesh induced by Joule heating. To that end, two vital modifications were proposed to the previously developed numerical method and compiled into a computation program. The first relies on the identification of the maximum temperature in the mesh, which relates to the criterion used to determine the melting of the mesh segment. The second modification relates to the resistivity of the metallic nanowire. As an example, the melting process of an Ag nanowire mesh was analyzed under specific working conditions. Numerical results allow monitoring of the temperature in the mesh under current stressing and determination of the current that triggers the melting of a mesh segment. Using the relationship between the melting current and the corresponding melting voltage, the electrical failure behavior of an Ag nanowire mesh system equipped with a current source can be predicted during actual operation.

## Methods

### Numerical model

**Figure 1:**
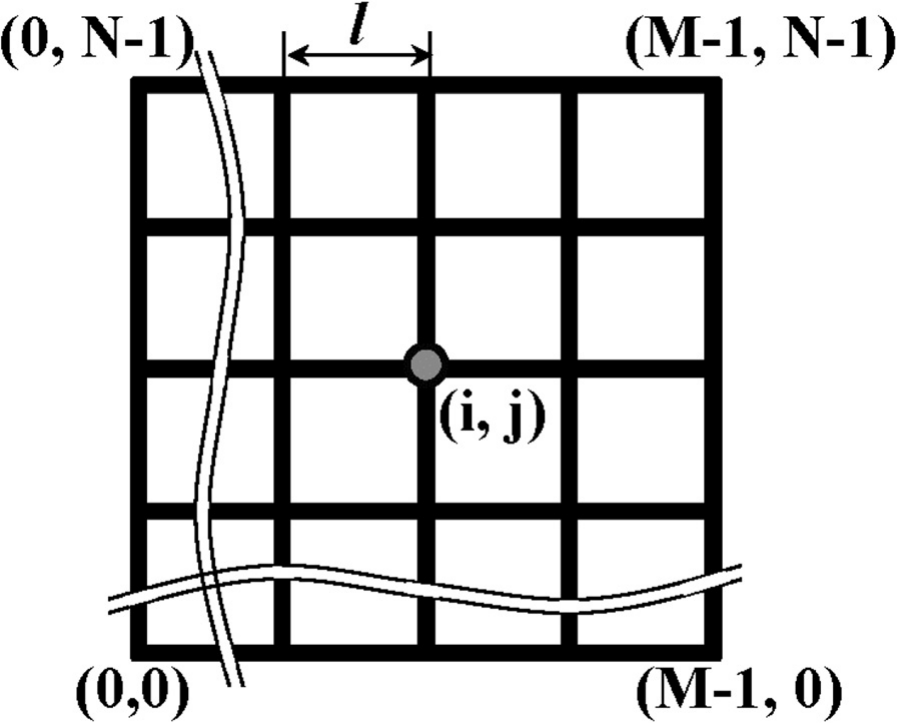
**Schematic illustration of a metallic nanowire mesh of dimension *M *× *N*.**

Figure 1 schematically illustrates a metallic nanowire mesh of dimension *M* × *N* that is a regular rectangular network with *M* columns and *N* rows. The pitch size of the mesh is *l*, and the cross-sectional area of the wire is *A*. The intersection of each row and column in the mesh is called a mesh node. Number the nodes by integral coordinates (*i*, *j*) (0 ≤ *i* ≤ *M*−1, 0 ≤ *j* ≤ *N* − 1), in which node (*i*, *j*) is the intersection of the (*i* + 1)th column and the (*j* + 1)th row. The corresponding number of mesh nodes is *M* × *N*.

The wire between two adjacent mesh nodes is called a mesh segment. The segment between node (*i* − 1, *j*) and node (*i*, *j*) is denoted by $S_{\left(i,j\right)}^{L}$, and the segment between (*i*, *j*) and (*i* + 1, *j*) is denoted by $S_{\left(i,j\right)}^{R}$. Similarly, the segment between node (*i*, *j* − 1) and (*i*, *j*) is denoted by $S_{\left(i,j\right)}^{D}$, and the segment between (*i*, *j*) and (*i*, *j* + 1) is denoted by $S_{\left(i,j\right)}^{U}$. Here, the letters L, R, D, and U denote the relative positions of the adjacent nodes (i.e., (*i* − 1, *j*), (*i* + 1, *j*), (*i*, *j* − 1) and (*i*, *j* + 1)) to node (*i*, *j*), meaning left, right, down, and up, respectively. The corresponding number of mesh segments is *M*(*N* − 1) + *N*(*M* − 1).

### Fundamentals of governing equations

The melting behavior of a metallic nanowire mesh can be treated as an electrothermal problem. To simplify this problem, the following assumptions are made: (1) the material of the metallic nanowire is electrically and thermally homogeneous and isotropic, (2) the material properties of the metallic nanowire are temperature independent, and (3) the effects of electromigration and corrosion are neglected.

**Figure 2:**
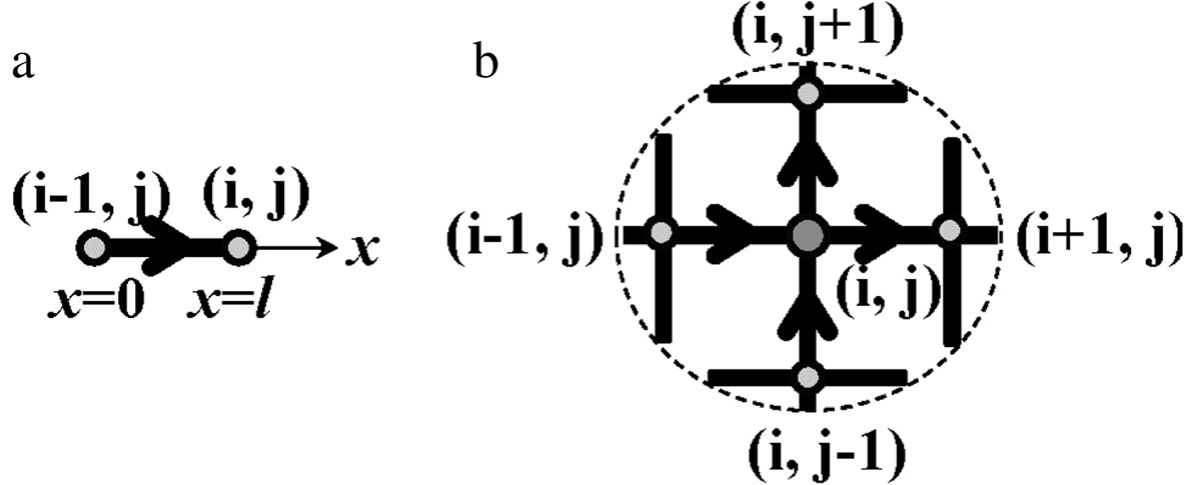
**Illustrations of (a) mesh segment**$S_{\left(i,j\right)}^{L}$**and (b) mesh node ( *i *, *j*).**

First, let us consider a mesh segment $S_{\left(i,j\right)}^{L}$ as a representative unit, whose surface is electrically and thermally insulated. As shown in Figure 2, current is input and output from nodes (*i* − 1, *j*), and (*i*, *j*), respectively. Using Ohm's law, the corresponding current density in the mesh segment $S_{\left(i,j\right)}^{L}$ can be calculated as

(1) $$j_{S_{\left(i,j\right)}^{L}}=-\frac{1}{\rho }\frac{\mathrm{d\phi}}{\mathrm{dx}}$$

Here, *ρ* is the electrical resistivity of the metallic nanowire, *ϕ* is the electrical potential, and *x* axis is along the axial direction of mesh segment (i.e., nanowire), which is rightward for lateral segment and upward for vertical one. Considering the heat conduction equation, we have

(2) $$\lambda \frac{d^{2}T}{dx^{2}}+\rho j_{S_{\left(i,j\right)}^{L}}^{2}=0$$

where *T* is the temperature and *λ* is the thermal conductivity of the nanowire. It should be noted that the effect of thermal conduction to the underlying substrate of the mesh is ignored here for simplicity.

At nodes (*i* −1, *j*) and (*i*, *j*) (i.e., at *x* = 0 and *x* = *l*), the temperatures are *T*_(*i*−1,*j*)_ and *T*_(*i*,*j*)_, respectively. Based on these boundary conditions, the temperature at any location of mesh segment $S_{\left(i,j\right)}^{L}$ can be obtained by solving Equation 2 as

(3) $$\begin{array}{l} T_{S_{\left(i,j\right)}^{L}}=-\frac{\rho }{2\lambda }j_{S_{\left(i,j\right)}^{L}}^{2}x^{2}+\left\{\frac{T_{\left(i,j\right)}-T_{\left(i‒1,j\right)}}{l}+\frac{\mathrm{\rho l}}{2\lambda }j_{S_{\left(i,j\right)}^{L}}^{2}\right\}x\\ \;\;+T_{\left(i‒1,j\right)} \end{array}$$

Using Fourier's law, the heat flux in the segment $S_{\left(i,j\right)}^{L}$ can be calculated as follows:

(4) $$\begin{array}{l} q_{{}_{S_{\left(i,j\right)}^{L}}}=-\lambda \frac{\mathrm{dT}}{\mathrm{dx}}\\ \;\;=\rho j_{S_{\left(i,j\right)}^{L}}^{2}x-\frac{\lambda }{l}\left\{T_{\left(i,j\right)}-T_{\left(i‒1,j\right)}\right\}-\frac{\mathrm{\rho l}}{2}j_{S_{\left(i,j\right)}^{L}}^{2} \end{array}$$

The current density, temperature, and heat flux in the other mesh segments connected to node (*i*, *j*) can be obtained in a similar manner.

Second, let us consider a mesh node (*i*, *j*). According to Kirchhoff's current law, we have

(5) $$I_{\mathrm{external}}+I_{\mathrm{internal}}=0$$

The term *I*_external_ represents the external input/output current at node (*i*, *j*), and *I*_internal_ represents the internal current at node (*i*, *j*), which is the sum of the currents passing through node (*i*, *j*) from the adjacent nodes. Note that the incoming current is positive and that the outgoing current is negative. In the present case, shown in Figure 2, we have

(6) $$I_{\mathrm{internal}}=\left\{j_{S_{\left(i,j\right)}^{L}}-j_{S_{\left(i,j\right)}^{R}}+j_{S_{\left(i,j\right)}^{D}}-j_{S_{\left(i,j\right)}^{U}}\right\}A$$

in which the subscript indicates the mesh segment connected to node (*i*, *j*) and *A* is the cross-sectional area of the wire. Considering Equations 1, 5, and 6 for any mesh node (*i*, *j*), a system of linear equations can be constructed to obtain the relationship between *ϕ* and *I*_external_ for any mesh node. Once *ϕ* is obtained for every node by solving the system of linear equations, the current density in any mesh segment can readily be calculated using Equation 1.

Similarly, according to the law of conservation of heat energy, we have

(7) $$Q_{\mathrm{external}}+Q_{\mathrm{internal}}=0$$

Here, *Q*_external_ represents the external input/output heat energy at node (*i*, *j*), and *Q*_internal_ represents the internal heat energy at node (*i*, *j*), which is the sum of the heat energy transferred through node (*i*, *j*) from the adjacent nodes. Note that the incoming heat energy is positive, and the outgoing heat energy is negative. In the present case, shown in Figure 2, we have

(8) $$Q_{\mathrm{internal}}=\left\{q_{S_{\left(i,j\right)}^{L}}-q_{S_{\left(i,j\right)}^{R}}+q_{S_{\left(i,j\right)}^{D}}-q_{S_{\left(i,j\right)}^{U}}\right\}A$$

Considering Equations 4, 7, and 8 for any mesh node, a system of linear equations can be constructed to obtain the relationship between *T* and *Q*_external_ for any mesh node. Once *T* is obtained for every node by solving the system of linear equations, the temperature at any location on any mesh segment can be calculated using Equation 3.

The current density and temperature in any mesh segment can be obtained using the previously described analysis for the electrothermal problem in a metallic nanowire mesh. This calculation will provide valuable information for the investigation of the melting behavior of a metallic nanowire mesh.

### Computational procedure

**Figure 3:**
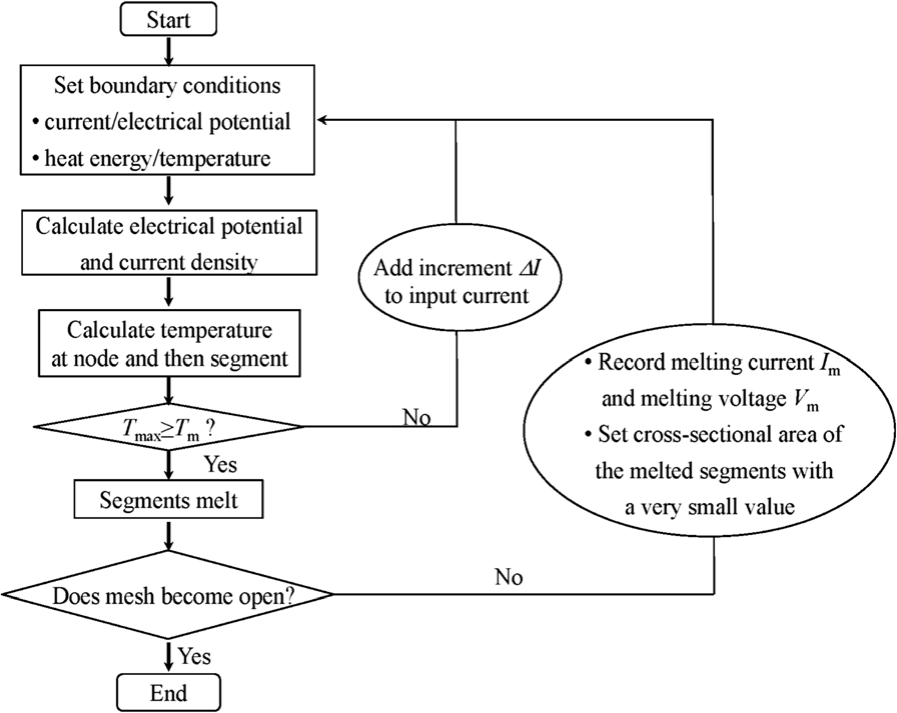
Flow chart of the computational procedure.

Based on the previously described analysis procedure, the as-developed computational program [24] was modified to investigate the Joule-heating-induced electrical failure of a metallic nanowire mesh. A flow chart of the program is shown in Figure 3.

Initially, the input current *I* is gradually increased in uniform increments, Δ*I*, and the corresponding temperature profile of the mesh is monitored. To cause the mesh segment to melt one at a time, Δ*I* must be properly tuned. When the temperature in a given mesh segment reaches the melting point *T*_m_ of the nanowire itself, the corresponding mesh segment melts and breaks with an arbitrary small force generated in actual operation such as a vibration. This temperature is considered the maximum temperature, *T*_max_, of the mesh. The electrical failure is believed to occur at the mesh segment. Here, the following two critical modifications have been made to the previously developed numerical method [24]. First, instead of using the temperature in the center of a mesh segment to approximate the *T*_max_, five points uniformly distributed along each segment are monitored to determine whether the temperature reaches *T*_m_ and melting occurs. If the temperature in a segment reaches *T*_m_ before the temperature at a mesh node, then the mesh segment melts and breaks. However, if the temperature of a mesh node reaches *T*_m_ first, then the adjacent segments connected to the node melt simultaneously and break. Second, the temperature dependence of the resistivity is ignored for simplification; thus, the resistivity of the metallic nanowire at the melting point, not the resistivity of the metallic nanowire at room temperature (R.T.), is employed during the simulation to approximate real conditions. The input current of the mesh triggering the melting of the mesh segment and the corresponding voltage of the mesh (i.e., the difference in the electrical potential between the input and the output) are recorded as the melting current *I*_m_ and the melting voltage *V*_m_, respectively. The corresponding resistance *R* of the mesh can be calculated by dividing *V*_m_ by *I*_m_.

Subsequently, the cross-sectional area of the melted mesh segment is set at a very small value to approximate a cross-sectional area of zero. The pathway of the current and heat in the mesh will be correspondingly renewed. By increasing the input current gradually, the current that triggers the subsequent melting of the mesh segment can be determined.

By repeating the aforementioned process until the mesh opens, the relationship between *I*_m_ and *V*_m_ can be determined throughout the melting process.

## Results and discussion

### Numerical model of an Ag nanowire mesh

**Figure 4:**
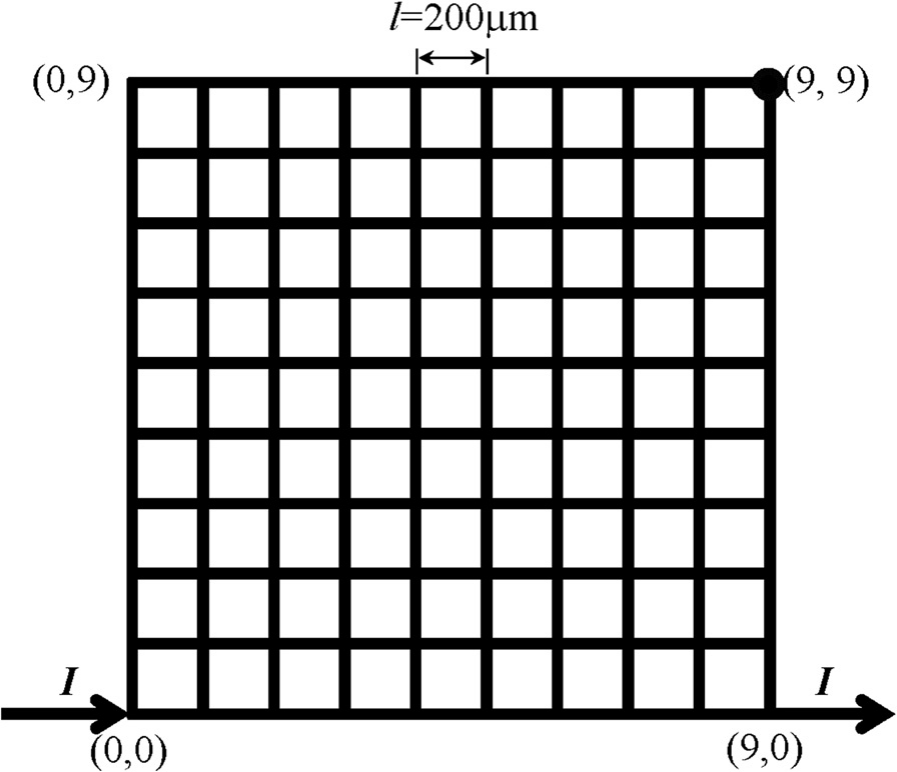
**Schematic illustration of an Ag nanowire mesh of size 10 × 10.**

An Ag nanowire mesh of size 10 × 10 is shown in Figure 4 as an example. The numbers of mesh nodes and mesh segments are 100 and 180, respectively. The pitch size is *l* = 200 μm, and the cross-sectional area of the Ag nanowire is *A* = 0.01 μm^2^. Taking into account the size effect, the physical properties of the Ag nanowire listed in Table 1 are employed in the simulation. Note that the melting point of Ag nanowire was experimentally measured to be 873 K [14]. The resistivity, *ρ*_m_, of the Ag nanowire at the melting point is estimated at 0.378 Ω∙μm using the resistivity, *ρ*_0_, of the Ag nanowire at R.T. and the temperature coefficient of resistivity, *α,* for bulk Ag.

**Table 1:** Physical properties of an Ag nanowire

**Physical properties**	**Value**
Melting point *T*_m_ (K)	873 [14]
Thermal conductivity at R.T. *λ* (W/μm∙K)	3.346 × 10^−4^[10]
Electrical resistivity at R.T. *ρ*_0_ (Ω∙μm)	0.119 [7]
Temperature coefficient of resistivity *α* (/K)	0.0038

In addition, the following working conditions are specified in the present study. The external current flows into the mesh from node (0, 0) and flows out of the mesh from node (9, 0), which means that node (0, 0) has an external input current and node (9, 0) has an external output current (see Figure 4). For all the other nodes, there is no external input or output current. A constant electrical potential is assigned to node (9, 9). The temperature of the boundary nodes ((*i*, 0), (0, *j*), (*i*, 9), (9, *j*) in which *i*, *j* = 0,…, 9) is set at room temperature of 300 K. For all of the other nodes, there is no any external input or output heat energy.

Using the developed computational program, the temperature in the Ag nanowire mesh can be monitored, allowing for determination of the melting current. The input current, *I*, is increased with a Δ*I* value of 0.001 mA to cause the mesh segments to melt one at a time if possible. The corresponding melting current and melting voltage (i.e., the difference in electrical potential between node (0, 0) and node (9, 0)) are recorded as melting current *I*_m_ and melting voltage *V*_m_, respectively. Using the relationship between *I*_m_ and *V*_m_, the variation in mesh resistance *R* throughout the melting process could be calculated.

### Numerical analysis of the failure behavior of the mesh

**Figure 5:**
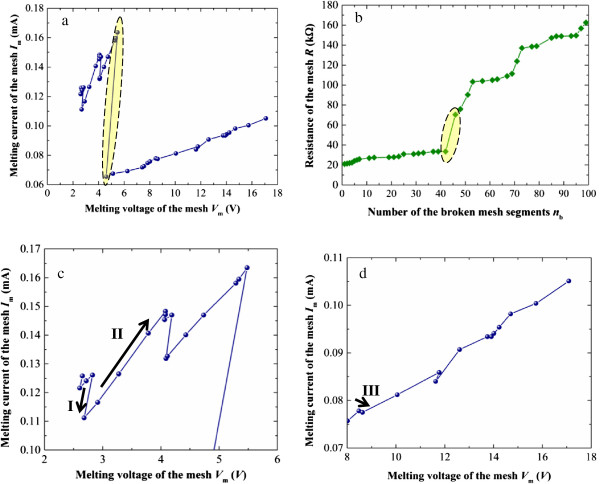
**Numerical analysis results for the melting process of the Ag nanowire mesh. (a)** Variation of the melting current and melting voltage, **(b)** variation of the mesh resistance, **(c)** starting stage, and **(d)** ending stage.

The as-obtained relationship between melting current *I*_m_ and melting voltage *V*_m_ and the calculated mesh resistance *R* versus the number of the broken segments during the whole melting process are shown in Figure 5a,b, respectively. To clearly observe the changing trend in *I*_m_, the starting stage and the ending stage of the melting process in Figure 5a are enlarged in Figure 5c,d, respectively. Although a repeated zigzag pattern is observed in the relationship between *I*_m_ and *V*_m_, *R* increases steadily during the melting process, in spite of the changing trend in *I*_m_.

**Figure 6:**
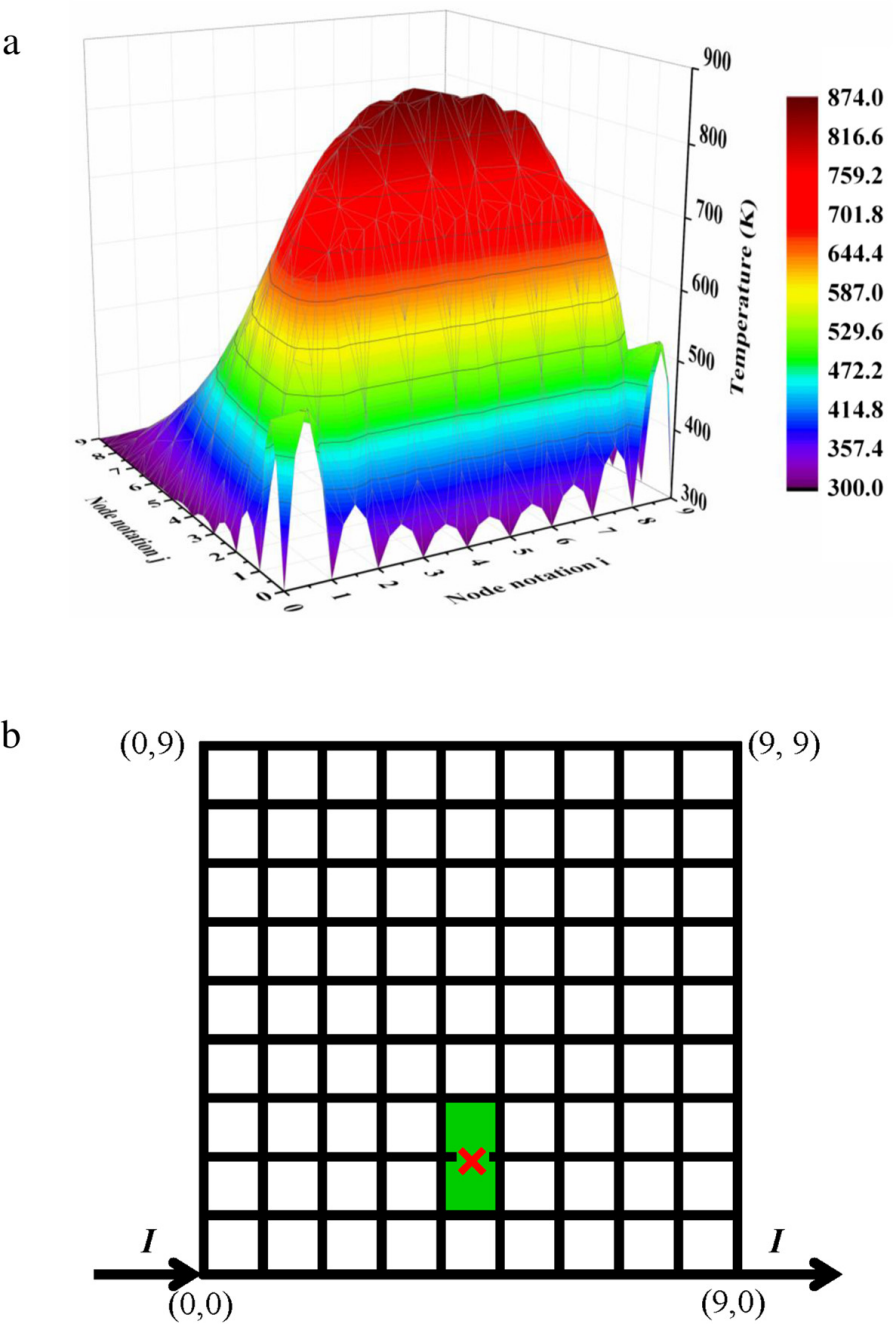
**Starting point of melting of the Ag nanowire mesh. (a)** Temperature profile and **(b)** mesh structure.

Initially, as the input current increases, the temperature of the mesh increases gradually. Moreover, the temperature at different locations of different segment should be different. When the maximum temperature in the mesh *T*_max_ reaches the melting point *T*_m_ of the nanowire, the corresponding mesh segment melts and breaks. This process is similar to the melting of an individual nanowire. As shown in Figure 5c, when the input current increases up to 0.126 mA, the Ag nanowire mesh starts to melt. The temperature profile of the mesh at this moment is shown in Figure 6a. The corresponding mesh structure is shown in Figure 6b, with the first melted segment marked by a red cross symbol.

Subsequently, the mesh structure undergoes a process of the consecutive melting of large numbers of individual nanowires. During the melting of the mesh as shown in Figure 5a, the variation in *I*_m_ and *V*_m_ of the mesh exhibits the repetition of three different trends: (I) both *I*_m_ and *V*_m_ decrease, (II) both *I*_m_ and *V*_m_ increase, and (III) *I*_m_ decreases while *V*_m_ increases. The solid-line arrows in Figure 5c,d indicate these three trends. Such repetition of zigzag pattern as shown in Figure 5a can be explained in detail as below. After one mesh segment is melted, the electrical pathway in the mesh is changed so that the mesh resistance increases, and therefore Joule heating increases. In one case, the maximum temperature of the mesh may be far beyond the melting point of the wire, which means the present current is much higher than that for the subsequent wire melting. To precisely obtain the melting current for the subsequent wire melting (i.e., the current when the maximum temperature of the mesh properly reaches the melting point), the input current has to be decreased, which means the decrease of melting current. In another case, the maximum temperature of the mesh is still lower than the melting point of the wire. To make further melting, the input current has to be increased to make the maximum temperature rise up to the melting point, which implies the increase of melting current. The irregular alternation of these two cases leads to the zigzag pattern of the relationship between *I*_m_ and *V*_m_ during the melting process of the mesh. Moreover, it is thought that if the pitch size of the mesh is smaller, the extent of zigzag pattern will be mitigated. In an extreme case, when the pitch size is zero which makes the mesh transit to thin film, the present zigzag pattern will be diminished and the relationship between *I*_m_ and *V*_m_ will become smooth.

**Figure 7:**
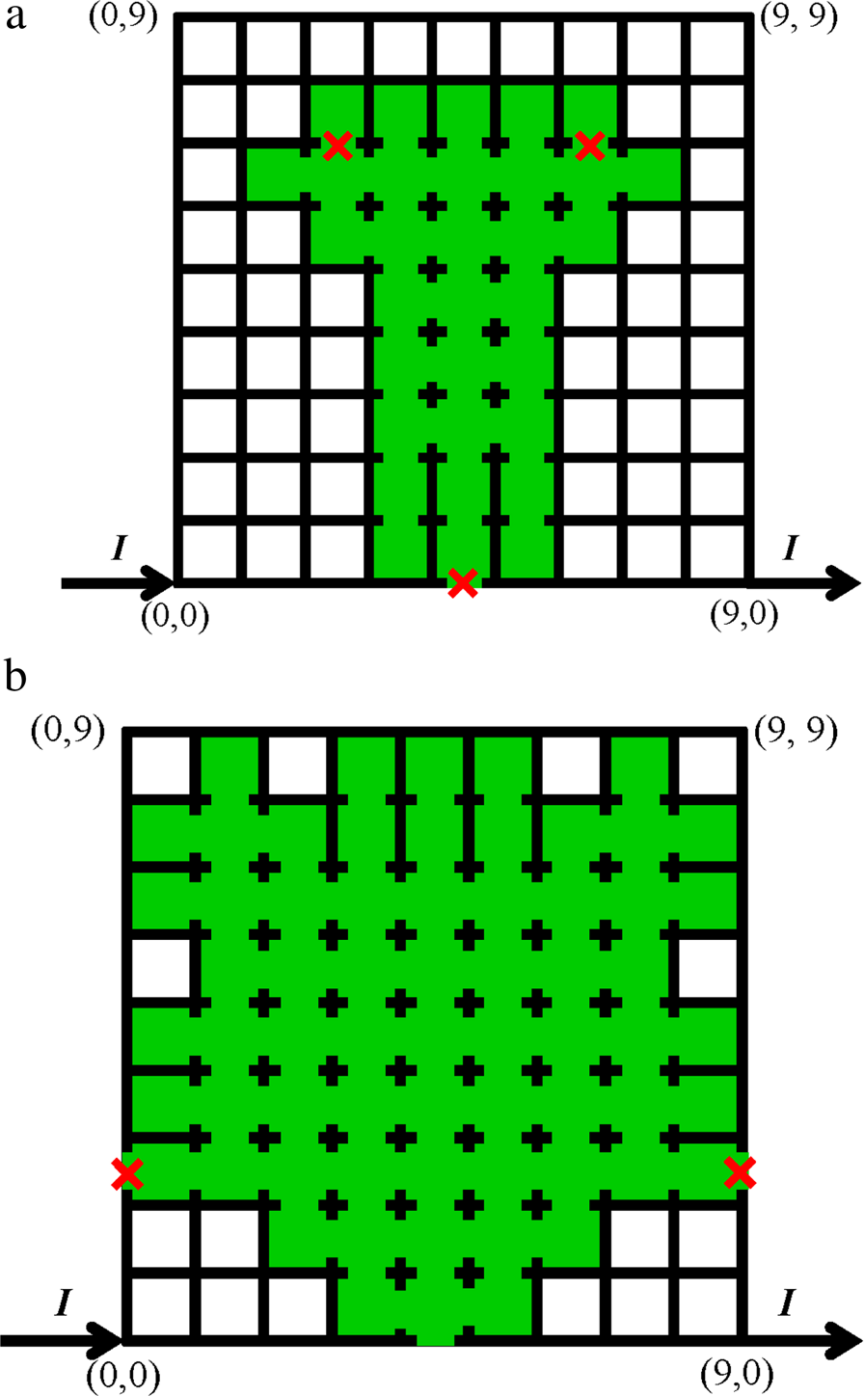
**Melting process of the Ag nanowire mesh. (a)** Mesh structure at the sudden fall of melting current and **(b)** mesh structure at the melting endpoint.

It is clear that there is a sudden sharp decrease in both *I*_m_ and *V*_m_ during the melting process (marked by an ellipse in Figure 5a), accompanied by a doubling of *R* (marked by an ellipse in Figure 5b). Although three segments melt simultaneously (marked by red cross symbols in Figure 7a), it is believed that the breakage of the segment located on the lower boundary of the mesh plays the key role by resulting in the detour of the current.

Finally, the mesh becomes open when two segments, marked by red cross symbols in Figure 7b, melt. Obviously, the broken mesh segments are sufficient to eliminate the continuous electrical pathway across the mesh. At this time, the total number of melted mesh segments is 99, which is slightly more than half the total number (180) of mesh segments in the intact Ag nanowire mesh.

The results of this work differ with those previously reported [24] in the following ways: First, the melting current is reduced by half, and the range of the melting voltage is increased, which can be attributed to the inclusion of *ρ*_m_. Second, any unreasonable drop in the melting current due to a possible numerical error has been removed. Third, throughout the melting process, the mesh remains symmetric regardless of the number of segments that melt, as shown in Figure 7. These results suggest a dramatic increase in the accuracy of numerical results, supporting the feasibility of the present modified numerical method.

### Prediction of the electrical failure behavior of the mesh equipped with current source

Achieving an immediate decrease in the current or voltage during practical experiments is known to be difficult due to the limited properties of current sources. Therefore, one cannot reproduce the above-mentioned zigzag pattern of *I*_m_ and *V*_m_ observed in the numerical melting process in actual experiments. Considering a system composed of an Ag nanowire mesh and a current source, the electrical failure behavior of the mesh in actual experiments could be predicted using the aforementioned numerical results. Two common modes of current sources, a current-controlled current source (CCCS) and a voltage-controlled current source (VCCS), are discussed below.

**Figure 8:**
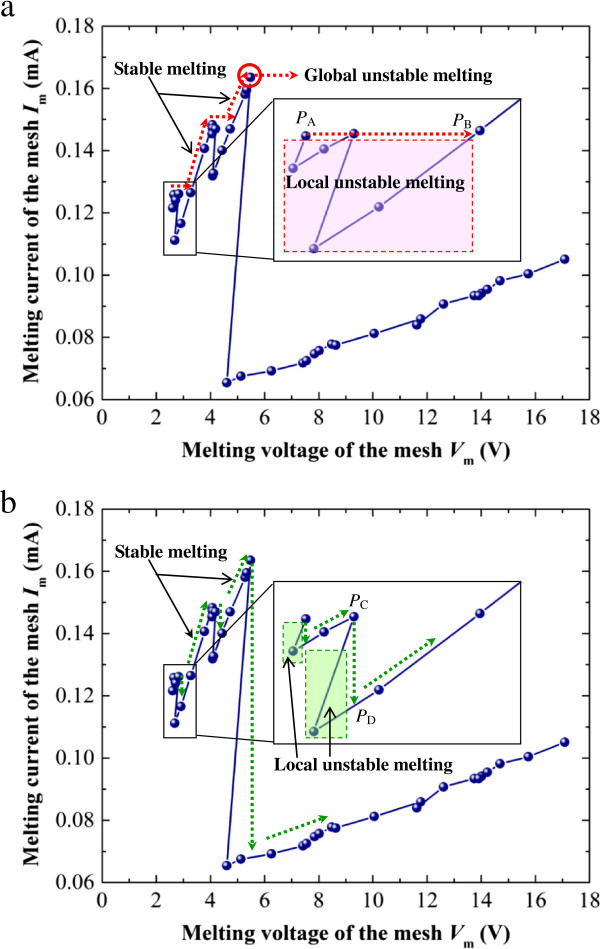
**Prediction of the melting of a real system containing Ag nanowire mesh with a current source. (a)** CCCS mode and **(b)** VCCS mode.

In the CCCS mode, the relationship between *I*_m_ and *V*_m_ of the mesh in a real experiment can be predicted as indicated in Figure 8a by the dotted-line arrows. The repetition of the platform stage is marked by the red dotted-line arrow pointing to the right, and the diagonal ascent stage is marked by the red dotted-lined arrow pointing up and to the right. The platform stage indicates the simultaneous melting of several mesh segments at a constant current, which is called local unstable melting. When compared to the curve of *I*_m_ vs. *V*_m_ produced in the numerical simulation of mesh melting, there is a jump (e.g., from point *P*_A_ to point *P*_B_ in the enlarged part of Figure 8a). The reason for this difference is that in real experiments, it is difficult to achieve an immediate decrease in the current. Therefore, it is difficult to reproduce the region at the lower side of the platform stage (i.e., the decrease in the current and the subsequent increase), which is marked by a red dashed rectangle in the enlarged part of Figure 8a. The diagonal ascent stage indicates that an increase in the current is necessary for the subsequent melting, which is called stable melting. It should be noted that when the current reaches the maximum, marked by a red open circle in Figure 8a, the mesh segments will melt simultaneously until the circuit of the mesh becomes open. This phenomenon, called global unstable melting, can be attributed to the increase in Joule heating, which is caused by the increase in the mesh resistance that accompanies the melting of the mesh segments.

Similarly, for the VCCS mode, the relationship between *I*_m_ and *V*_m_ of the mesh in a real experiment can be predicted as indicated in Figure 8b by the dotted-line arrows. The repetition of the vertical decline stage is marked by a green dotted-line arrow pointing downward, and the diagonal ascent stage is marked by a green dotted-line arrow pointing up and to the right. The vertical decline stage indicates the simultaneous melting of several mesh segments at a constant voltage. This local unstable melting is similar to the local unstable melting that occurs in the CCCS mode. When compared to the curve of *I*_m_ vs. *V*_m_ during numerically simulated melting, there is a jump (e.g., from point *P*_C_ to point *P*_D_ in the enlarged part of Figure 8b). The reason for this jump is that in real experiments, it is difficult to decrease the voltage immediately, just as it is difficult to decrease the current immediately. Therefore, it is difficult to reproduce the region to the left side of the vertical decline stage (i.e., the decrease in voltage and its subsequent increase), which is marked by a green dashed rectangle in the enlarged part of Figure 8b. The diagonal ascent stage indicates that an increase in the voltage is necessary for further melting. This stable melting is also similar to the stable melting that occurs in the CCCS mode. However, no global unstable melting occurs as in the CCCS mode due to the decrease in Joule heating, which is caused by the increase in the mesh resistance that accompanies the melting of the mesh segments.

To fully understand the unique melting behavior of a metallic nanowire mesh, the melting behavior of an individual nanowire itself is summarized for comparison as follows: For both the CCCS and VCCS modes, once the maximum temperature in the nanowire reaches *T*_m_, the nanowire melts and breaks. This behavior has been used to cut metallic nanowires at desired locations [15,17].

The predicted stable and unstable melting in the Ag nanowire mesh equipped with a current source is only an example. In the present case, the thermal conduction to the underlying substrate of the mesh is ignored. According to the above analyses, it could be speculated that the melting current *I*_m_ and the corresponding melting voltage *V*_m_ will increase if the effect of the underlying substrate is taken into account. The reason is the thermal conduction to substrate can effectively mitigate the temperature rise. However, as thermal conduction to the substrate is a global effect, the mesh itself including all mesh segments will be affected. Therefore, the overall zigzag behavior of the mesh and the predicted stable/unstable melting may not be changed largely.

Moreover, it should be noted that there are some important parameters including boundary conditions (e.g., thermal conduction to substrate), mesh structure, electromigration, and corrosion, all of which will make a great effect on the electrical failure behavior of metallic nanowire mesh due to Joule heating. The present study just provides a basis for investigating the reliability of metallic nanowire mesh.

## Conclusions

With a modified effective computational method in terms of the maximum temperature in the mesh and the electrical resistivity, the electrical failure of a metallic nanowire mesh due to Joule heating (i.e., melting) was investigated. As an example, the melting process of an Ag nanowire mesh under specific working conditions was analyzed via monitoring of the temperature in the mesh and determining the melting current that triggers the melting of a mesh segment. Using the as-obtained relationship between the melting current and the corresponding melting voltage during the melting process, the real melting behavior of a mesh system equipped with a current source could be predicted. The corresponding numerical results indicate with high accuracy that local unstable and stable melting can be identified in both current-controlled and voltage-controlled current sources in the present example.

## Abbreviations

ITO: Indium tin oxide; CCCS: Current-controlled current source; VCCS: Voltage-controlled current source.

## Competing interests

The authors declare that they have no competing interests.

## Authors’ contributions

YL, KT, and MS participated in the design of the study and the analysis of its results. Discussion and revision were from HT and MS. YL drafted and finalized the manuscript. All authors read and approved the final manuscript.
